# Decreased food intake as a fecundity-dependent cost of reproduction in keelback snakes (*Tropidonophis mairii*, Colubridae)

**DOI:** 10.1098/rsos.241831

**Published:** 2025-04-09

**Authors:** Gregory Paul Brown, Richard Shine

**Affiliations:** ^1^ School of Natural Sciences, Macquarie University, Sydney, New South Wales, Australia

**Keywords:** life-history evolution, Natricinae, reproductive effort, tropical ecology

## Abstract

The physical burden of pregnancy may render females slower and less able to evade predation, favouring a reduction in feeding in order to avoid a reduction in survivorship. Life-history theory predicts that an organism’s optimal level of investment into reproduction depends upon whether or not the associated ‘costs’ (such as a decrease in rate of feeding) increase with higher fecundity. Anorexia during pregnancy is widespread among snakes, but there are few field data on fecundity-dependence of such costs. Over a 23-year period, we recorded reproductive condition and feeding status (based on palpation and production of faeces) for 3778 captures of free-ranging female natricine colubrid snakes (keelbacks, *Tropidonophis mairii*) in tropical Australia. Pregnancy reduced feeding rates, and that decrease was greatest for females with higher reproductive investment (clutch mass relative to maternal mass). Our long-term data provide the first clear-cut evidence of fecundity-dependent costs of reproduction in free-ranging snakes.

## 1. Introduction

An organism’s reproductive output is a primary determinant of its fitness in evolutionary terms, and population-wide averages in reproductive output are critical to population viability [1–3]. That dual significance has stimulated biologists to document variation in reproductive output, and search for adaptive explanations for that variation [4]. Life-history models predict that optimal values for reproductive output are determined by the balance between costs and benefits of higher fecundity. The benefits of larger brood sizes for maternal fitness are clear (i.e. more offspring = higher fitness) [5], but costs are more difficult to quantify and may differ among study systems. For example, in snakes that live in arid environments a large clutch may increase rates of water loss because of stretching of the skin between the scales [6]. In cool environments, pregnant females of heliothermic taxa may be sedentary in sun-exposed microhabitats where they can thermoregulate precisely and thereby accelerate embryonic development [7–9]. One generally important cost may be that a large clutch imposes a physical burden that renders a reproducing female less agile or athletic, increasing her vulnerability to predation [10–14]. In response to such costs, females of many species curtail feeding when they are burdened by developing offspring [15,16].

Snakes have been popular study organisms with which to explore the impact of pregnancy on rates of feeding, because their low metabolic rates enable them to withstand long periods of anorexia [17]. In many snake species, gravid females forego foraging and instead remain sedentary and thermoregulate precisely [18]. In some cases, the distended body of a pregnant squamate may impair their locomotion and thus foraging ability [14,19]. In some snake taxa, however, feeding continues throughout pregnancy, and easy availability of prey (such as in captivity) can stimulate feeding during pregnancy in some (but not all) species in which gravid females rarely feed in the wild (see review by [17]). In a few species, geographic and/or temporal variation in feeding by gravid females has also been documented [20,21]. However, the impact of level of reproductive investment on feeding by gravid females has not been examined in detail for any population of snakes (see [22] for an example with lizards). In the current paper, we use data from a long-term field study on tropical natricine snakes to assess the extent to which female reproductive investment compromises rates of feeding and locomotor ability. Specifically, we asked:

(1) Does pregnancy reduce rates of feeding (i.e. comparing gravid to non-gravid adult female snakes)?
(2) Does a female’s level of reproductive investment (relative clutch mass) influence her feeding rate?

## 2. Methods

### 2.1. Study species

**Figure 1.**
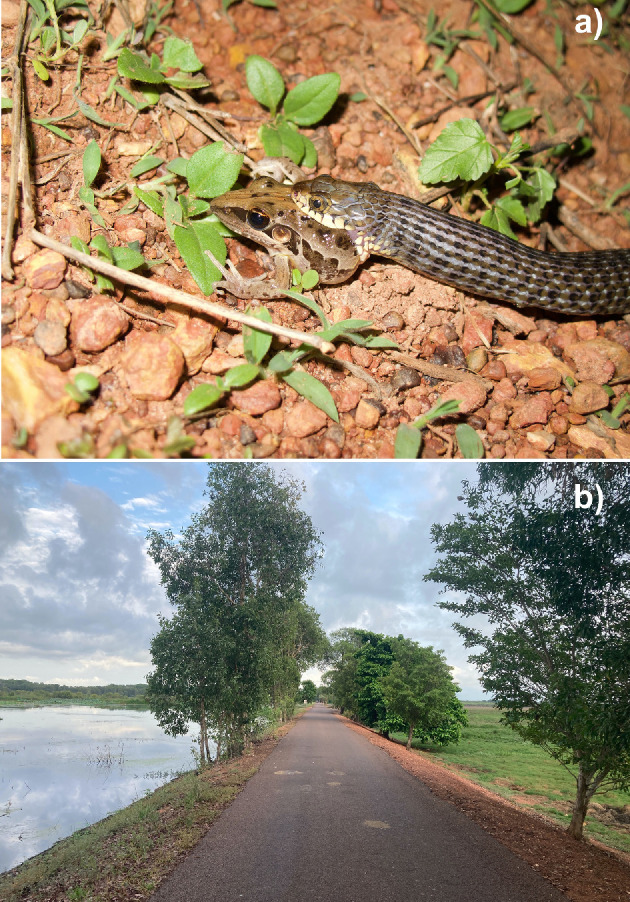
(a) A small adult female keelback (*T. mairii*) consuming a frog (*Litoria nasuta*), and (b) the roadway atop the wall of Fogg Dam, our study site. Photographs by G.P.B.

Keelbacks (*Tropidonophis mairii*; figure 1a) are the sole Australian representative of the widespread colubrid subfamily Natricinae (accorded familial status by some researchers). These rugose-scaled swamp-dwellers can attain maximum adult body sizes (snout–vent length; SVL) of 82 mm (females) and 68 mm (males), are crepuscular and nocturnal, and feed primarily on frogs [23,24]. Females produce up to three clutches of 3–20 eggs per annum during the tropical dry season. Maturation can occur at less than 12 months of age [25]. Previous research on our study population has documented reproductive biology in detail [25,26] and has shown that pregnancy reduces locomotor speeds; that gravid female keelbacks flee from an approaching human at greater distances than do non-gravid females [11]; and that females produce larger clutches relative to maternal body size in years when frogs are more abundant [27].

### 2.2. Study area

Fogg Dam (figure 1b) is an artificial impoundment on the floodplain of a tributary of the Adelaide River, 60 km east of the city of Darwin (12.56° S, 131.27° E). The area experiences a wet–dry climate, with rainfall concentrated in the months from December to March (mean annual rainfall over this period is 1080 mm, out of an annual total of 1375 mm). Temperatures are high year-round (mean monthly maximum air temperatures exceed 30°C every month). Keelbacks that forage in surrounding riparian habitats migrate to the higher drier soil of the dam wall for oviposition [11,28].

### 2.3. Methods for data collection

During the April–July nesting seasons from 1998 to 2021, one of us (G.P.B.) walked the wall of Fogg Dam in the early evening to collect snakes. These were captured by hand, placed in individual cloth bags and returned to our nearby field laboratory for processing and measuring for SVL and mass. Data on feeding included observations made during capture, such as prey consumption (figure 1a), or prey items regurgitated in holding bags or during handling. During handling, we gently palpated the snake’s abdomen in a posterior direction to void any faeces in the intestine (and record its presence) and to detect and count shelled eggs. Snakes were individually marked by scale-clipping and released within 24 h of capture at their initial capture location, with the exception of females containing shelled eggs. These gravid females were retained in captivity and held in individual cages containing a water dish and nest box lined with damp vermiculite. After they laid their eggs, typically within 7 days of capture, the females were re-weighed and then released back at their original capture location. Because we recorded body masses of females at capture and after oviposition we were able to quantify mass loss and hence calculate relative clutch mass (RCM) as mass loss divided by postpartum body mass [29,30].

### 2.4. Methods for statistical analysis

#### 2.4.1. Does pregnancy affect feeding rates?

Our first analysis compared feeding rates of adult-sized (>44.75 mm SVL) females captured during April to July, the months when 90% of nesting occurs. This comparison was based on 3778 captures of 3450 individual female snakes. The data were analysed using a mixed model with presence of food/faeces (Yes versus No) as the dependent variable with a binary distribution and a logit link function. Independent variables were reproductive status (gravid, Yes versus No) and SVL was included as a covariate. Year was included as a random effect to represent annual variation in prey availability and climate, and we used an autoregressive error term to overcome potential statistical artefacts associated with autocorrelation (i.e. in case consecutive years were similar).

#### 2.4.2. Does reproductive investment affect the rate of feeding?

Our second analysis of factors affecting the probability of containing food or faeces was based on 1160 captures of gravid females (1088 individuals) for whom we calculated RCM. All these females oviposited in captivity within 10 days of capture, thus feeding records correspond to the last stages of pregnancy. Our analysis used a mixed model analysis (as above) with presence of food (Yes versus No) as the dependent variable, and SVL and RCM as independent variables. We again included year as a random effect and used an autoregressive error term as above to reduce potential autocorrelation between years.

All analyses were conducted using the Glimmix procedure in SAS 9.4 (SAS Institute, Cary, NC, USA). We inspected residual plots to check for normality and heterogeneity of error distributions.

## 3. Results

### 3.1. Does pregnancy affect feeding rates?

**Figure 2.**
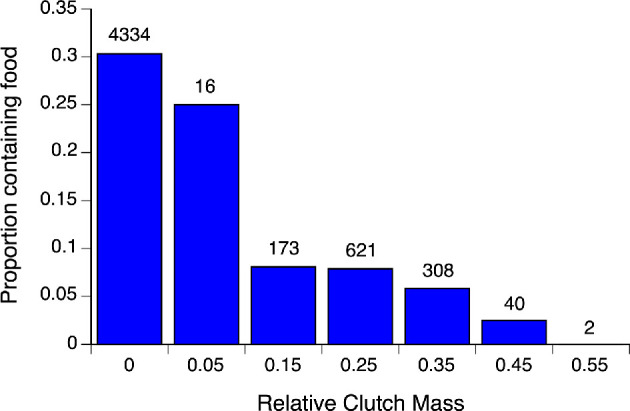
Effect of pregnancy (presence of oviductal eggs) on rates of feeding in female keelback snakes (*T. mairii*). The figure shows the proportion of snakes containing recently ingested prey as a function of reproductive condition. Non-gravid snakes are shown as relative clutch mass (RCM) = 0; all other categories are bin midpoints of 0.10 RCM width (e.g. >0 to 0.10; >0.10 to 0.20, etc.). Numbers above histograms show sample sizes (number of occasions on which a snake’s feeding status was determined).

Of the 2382 captures of non-gravid females during the nesting season, 707 (30%) of animals contained food or faeces. Of 1396 captures of gravid females, only 86 (6.2%) contained food or faeces. The mixed model analysis indicated that reproductive status was a significant predictor of feeding status (*F*_1,3752_ = 158.18, *p* < 0.0001; table 1), with gravid females significantly less likely to have eaten (figure 2). Unambiguous signs of recent feeding (e.g. captured while feeding, regurgitated prey, palpable prey in stomach) were less common than feeding inferred by the presence of faeces, but the rarity was similar in reproductive and non-reproductive females (1.0% versus 3.8% of observations, respectively, *χ* ^2^ = 2.1, *p* = 0.15).

**Table 1.** Multivariate analyses of the effects of (i) female body size (SVL) and reproductive status (gravid, Yes/No) on feeding (food present, Yes/No) and (ii) female body size and reproductive investment (as measured by relative clutch mass, RCM) on feeding (food present, Yes/No). Bold text indicates *p* < 0.05.

dependent variable	independent variables	d.f.	estimate	*F*	*p*
(i) food present Y/N	SVL	1,3752	−0.011	2.16	0.1417
	gravid Y/N	1,1752	1.67	158.18	**<0.0001**
(ii) food present Y/N	SVL	1,1134	0.001	0.01	0.9431
	RCM	1,1134	−4.237	6.89	**0.0088**

### 3.2. Does reproductive investment of gravid keelbacks affect the rate of feeding?

For the 1160 captures of gravid female keelbacks for which we calculated reproductive investment, 86 (7%) contained food/faeces. RCM of gravid snakes ranged from 0.027 to 0.583. The likelihood that a gravid female would contain food/faeces was negatively related to her RCM (*F*_1,1134_ = 6.89, *p* = 0.0088; table 1), and the mean RCM of gravid females that contained food was lower than that of females that did not contain food (24.3% versus 26.9%, *F*_1,1158_ = 10.60, *p* = 0.0012; figure 2).

## 4. Discussion

Free-ranging gravid female keelbacks fed less often than did non-gravid conspecifics; and females with higher reproductive investment (RCM) fed less often than did conspecifics with lower RCM. These patterns cannot be attributed to short-term events (because we gathered the data over a long time period) or differences in mean body size between reproductive and non-reproductive snakes (because we included maternal body size as a covariate in our analyses). Neither can the patterns be driven by spatial or temporal variation in factors such as prey availability (because we collected reproductive and non-reproductive animals at the same times and in the same places and included year as a random factor in our analyses). In short, our unusually large long-term dataset provides robust evidence that pregnancy reduces feeding rates in keelbacks and does so in a fecundity-dependent manner.

### 4.1. Why do gravid keelbacks feed less often than non-gravid ones?

Many authors have attributed anorexia of reproductive snakes to the incompatibility between foraging and the precise thermoregulation that is required to enhance offspring viability [17]. This mechanism may be important for diurnal sun-basking taxa, but is not applicable to our tropical study species because keelbacks in our study area do not bask, and are rarely active by day [11,25,28]. Likewise, the idea that space for prey in the stomach is reduced by the distension from oviductal eggs or embryos is unlikely to be important for snakes because of their elongate body plan; the oviducts overlap only minimally with the stomach [17]. The most plausible advantage of reduced feeding, then, is to reduce a female’s exposure to predators at a time when she is slowed by the mass and volume of her clutch. In keeping with that hypothesis: (i) wading birds consume many snakes on the wall of Fogg Dam [31] and (ii) gravid keelbacks flee from an approaching human at greater distances than do non-gravid conspecifics [28].

A trend for pregnancy to reduce feeding rates has been documented in many (but not all) other studies of snakes (review by [17]; see also [20,21]). In almost all cases, the results involve a simple comparison between rates of feeding in reproductive versus non-reproductive animals. Such analyses confirm that a reduced rate of prey consumption is a widespread cost of reproduction in female snakes. However, to bring that result to bear on theoretical (mathematical) models for life-history evolution, we also need to know if the magnitude of that cost is affected by a female’s level of investment into reproduction. No previous study on snakes has had the sample size needed to explore that issue.

### 4.2. Why does low reproductive investment increase rate of feeding?

Mathematical models for life-history evolution posit that the relationship between reproductive output and costs (such as decreased rates of feeding) is critical. If the cost is independent of fecundity (i.e. all pregnant females cease feeding), then selection is expected to favour high reproductive expenditure per bout even if this entails low frequency of reproduction [32], that is, the optimal life-history tactic is to pay the cost as infrequently as possible. In contrast, fecundity-dependence of costs (feeding rates decrease as reproductive investment increases) may favour frequent production of smaller-than-maximal clutches [32].

Costs of reproduction can be assessed in two currencies: survival or energy balance [33]. In practice, survival costs of reproduction are difficult to measure in the field because:

(1) mortality events are difficult to observe directly, creating uncertainty about causes of an individual’s disappearance from the population. That disappearance might be due to dispersal or inactivity rather than mortality; and even if the individual has died, the cause may be unrelated to predation, or specifically to locomotor decrements imposed by reproductive investment;
(2) relationship among relevant variables may be highly nonlinear. For example, even a massive decrement in mobility may not affect vulnerability to predation if predators can capture even the fastest-moving individuals [34], or if predators are unable to capture even the slowest-moving individuals. In such cases, laboratory measures of locomotor performance will not predict an individual’s vulnerability to mortality; and
(3) females can modify their behaviour in a way that translates a survival ‘cost’ into an energy cost, by foregoing feeding and, instead, staying in safe refuges that are inaccessible to predators [35].

The costs and benefits of anorexia may offer additional perspective on the patterns we observed. A negative energy balance is the cost of feeding cessation. However, this cost may be overshadowed by the benefit of reduced predation risk while foraging. This benefit would be magnified in heavily gravid females whose reproductive value is extremely high [36]. If females with higher RCM experience higher predation risk because they are more detectable to or less able to escape a predator, they may benefit more from anorexia than females with lower RCM.

Effects of reproduction on rates of feeding are easier to quantify in the field than are survival costs. We simply need a measure of food consumption (such as stomach contents or faecal output) that can be compared among reproductive versus non-reproductive individuals and can be compared to some measure of reproductive investment within the former group. This is the approach taken in the current paper.

A lack of prior field studies of fecundity-dependence of reproductive costs makes it difficult to compare our data to other work. Several laboratory-based projects have reported that RCM predicts the magnitude of locomotor impairment during pregnancy [37–39] and the total metabolic expenditure of a gravid female [16,40] (but see [41] for a different conclusion). As noted above, however, these measurements are difficult to translate into the field. For example, Stahlschmidt *et al*. [42] found that egg-brooding pythons (*Liasis fuscus*) with larger clutches maintained lower incubation temperatures, eliminating the expected increase in total metabolic expenditure with increased clutch size. In a study of amphibious sea kraits (Laticaudinae), Brischoux *et al*. [17] reported a progressive decline in feeding frequency as ovarian follicles increased in size. The consequent relationship between total reproductive burden (follicle mass) and food intake resembles a fecundity-dependent cost (more burden = less feeding) but might be driven by endocrine shifts as follicles mature, rather than by among-individual variation in reproductive expenditure. To our knowledge, the only quantitative analysis of the relationship between inter-individual variation in reproductive investment versus costs was the Bonnet *et al*. [43] mark–recapture study on vipers (*Vipera aspis*); those authors reported that survival of females was not related to their fecundity. In the same species, relative litter mass was not correlated with a female’s growth rate, but was positively correlated with her rate of mass recovery postpartum (rather than negatively related, as expected if higher reproductive investment was a cost to recovery) [44].

The ecological diversity of snakes erodes the possibility of any universal patterns in reproductive costs, or in their fecundity-dependence. It is already apparent that pregnant females cease feeding in some species but not others, and that prey availability can influence the magnitude of anorexia (see Brischoux *et al*. [17] and references therein). Likewise, the relationship between reproductive investment and associated costs doubtless varies among species, and even through time (based on prey abundance) in any given system. Some components of reproductive costs likely are independent of fecundity (e.g. the risks and energy costs of migrating to a nesting site) whereas others (such as the reduction in feeding in gravid keelbacks) may increase with higher investment into reproduction. Long-term mark–recapture studies, such as our own, may provide the best opportunity to assess the nature of such costs. Proximate effects of feeding during litter development in snakes also warrant further study. In some cases, supplemental feeding during pregnancy can adversely affect reproduction in rattlesnakes, though the mechanisms are unclear [45].

## Data Availability

Data are available from the Dryad data repository [46].
